# Nanoparticle and Nanostructure Synthesis and Controlled Growth Methods

**DOI:** 10.3390/nano12183226

**Published:** 2022-09-16

**Authors:** Vancha Harish, Md Mustafiz Ansari, Devesh Tewari, Manish Gaur, Awadh Bihari Yadav, María-Luisa García-Betancourt, Fatehy M. Abdel-Haleem, Mikhael Bechelany, Ahmed Barhoum

**Affiliations:** 1 School of Pharmaceutical Sciences, Lovely Professional University, Phagwara 144411, Punjab, India; 2 Department of Pharmacognosy and Phytochemistry, School of Pharmaceutical Sciences, Delhi Pharmaceutical Sciences and Research University, New Delhi 110017, India; 3 Centre of Biotechnology, University of Allahabad, Prayagraj 211002, Uttar Pradesh, India; 4 Centro de Investigaciones Químicas, IICBA, Universidad Autónoma del Estado de Morelos, Cuernavaca 62209, Mexico; 5 Chemistry Department, Faculty of Science, Cairo University, Giza 12613, Egypt; 6 Center for Hazards Mitigation, Environmental Studies and Research (CHMESR), Cairo University, Giza 12613, Egypt; 7 Institut Europeen des Membranes, IEM, UMR 5635, University of Montpellier, ENSCM, CNRS, 34730 Montpellier, France; 8 NanoStruc Research Group, Chemistry Department, Faculty of Science, Helwan University, Cairo 11795, Egypt; 9 School of Chemical Sciences, Dublin City University, D09 Y074 Dublin, Ireland

**Keywords:** nanomaterials dimensionality, synthesis approaches, reaction phase, mechanical synthesis, physical synthesis, chemical synthesis, physicochemical synthesis, biological synthesis, growth-controlled mechanisms

## Abstract

Nanomaterials are materials with one or more nanoscale dimensions (internal or external) (i.e., 1 to 100 nm). The nanomaterial shape, size, porosity, surface chemistry, and composition are controlled at the nanoscale, and this offers interesting properties compared with bulk materials. This review describes how nanomaterials are classified, their fabrication, functionalization techniques, and growth-controlled mechanisms. First, the history of nanomaterials is summarized and then the different classification methods, based on their dimensionality (0–3D), composition (carbon, inorganic, organic, and hybrids), origin (natural, incidental, engineered, bioinspired), crystal phase (single phase, multiphase), and dispersion state (dispersed or aggregated), are presented. Then, the synthesis methods are discussed and classified in function of the starting material (bottom-up and top-down), reaction phase (gas, plasma, liquid, and solid), and nature of the dispersing forces (mechanical, physical, chemical, physicochemical, and biological). Finally, the challenges in synthesizing nanomaterials for research and commercial use are highlighted.

## 1. Introduction

Nanomaterials (NMs), nanochemistry, nanotechnology, and nanoscience are just some of the new terms in the nanotechnology field that frequently appear in journals of materials science, engineering, and medicine, as well as in popular books and newspapers, and are becoming known by a wide audience, including laypersons [1,2]. NMs are materials with one or more nanometric dimensions (internal or external) (i.e., 1–100 nm) [3]. NMs can be divided into different groups in the function of their dimensionality (0–3D), morphology (low and high aspect ratio), porosity (macro-, nano-, mesoporous), composition (carbon, inorganic, organic, and hybrids), origin (natural, incidental, engineered, bioinspired), phase (single phase, multiphase), and dispersion state (dispersed or aggregated) [4,5]. The synthesis methods of NM are generally classified into three categories: (i) liquid phase synthesis methods (the most important), in which biological or biochemical processes are carried out in solution; (ii) gas phase synthesis methods based on NM nucleation, growth, and deposition under vacuum or plasma phase conditions; and (iii) solid phase synthesis, including grain formation and alloying [6]. The nanoparticles are synthesis methods that can be further classified into three main types, namely physical, chemical, and biological processes that have been a vast improvement over time to include physiochemical, and mechanochemical processes.

Humans have always prepared and used NMs and nanocomposites [7]. Recent discoveries show that NMs were developed in different eras (from prehistory to contemporary times). *Homo sapiens* have been producing carbon-based NMs since the discovery of fire. Carbon NMs (composite of fat, charcoal, and pigments) were discovered in hand stencils in Sulawesi caves (Indonesia) from 40,000 BCE (according to carbon dating results) [8]. Ancient Egyptians (4000 years ago) produced hair dyes using PbS nanoparticles with a size of ~5 nm produced by chemical synthetic processes. Inorganic NMs were first produced in Egypt and Mesopotamia in the fourteenth and fifteenth centuries BC, and other inorganic materials were used to make glassware [9]. In 1959, Feynman first introduced the modern concepts of nanotechnology, nanoscience, and nanomaterials and their significance (“There’s Plenty of Room at the Bottom”) [10,11,12]. Nanoscience takes us deeper because it immerses us in the study and discussion of scientific phenomena and the fundamental nature of molecules and compounds smaller than 100 nm. To date, there are several important applications of nanomaterials such as water treatment [13], hydrogen production, fuel cells [14], batteries [15], sensors [16,17], diagnosis [18], and drug delivery [19].

Various synthesis methods can be used to produce NMs, which affect their size, shape, and surface functionality [20]. NMs can be produced using “bottom-up” synthesis methods (from molecules/atoms in the solid, liquid, or gaseous phase to nanoparticles) or “top-down” approaches (from bulk material). There have been many techniques and applications reported in the last five years. This review provides an overview of NMs, including their history, definitions, and unique properties. Different classifications of nanomaterials based on their size, shape, origin, compositions, and other specific features are presented systematically. The various NM classifications, synthesis approaches, functionalization techniques, and growth-related mechanisms are discussed. First, the different classifications of NMs (based on their size, composition, origin, crystal phase, and dispersion state) are presented. Then, the synthesis methods are classified and discussed in function of the starting materials, reaction phase, and nature of the dispersing forces. The basic concepts of nanoparticle formation mechanisms and growth control. Finally, the challenges associated with the synthesis of NMs for research and commercial purposes are highlighted. This review provides fundamental insight for readers into the properties of various classes of nanomaterials and their synthesis methods in one place.

## 2. Classification of Nanomaterials

NMs can be classified into four broad types, regardless of their location or chemical properties.

### 2.1. Classification Based on Dimensionality

In 2007, Pokropivny and Skorokhod proposed a novel NM categorization method based on their dimensionality [21].

**Figure 1:**
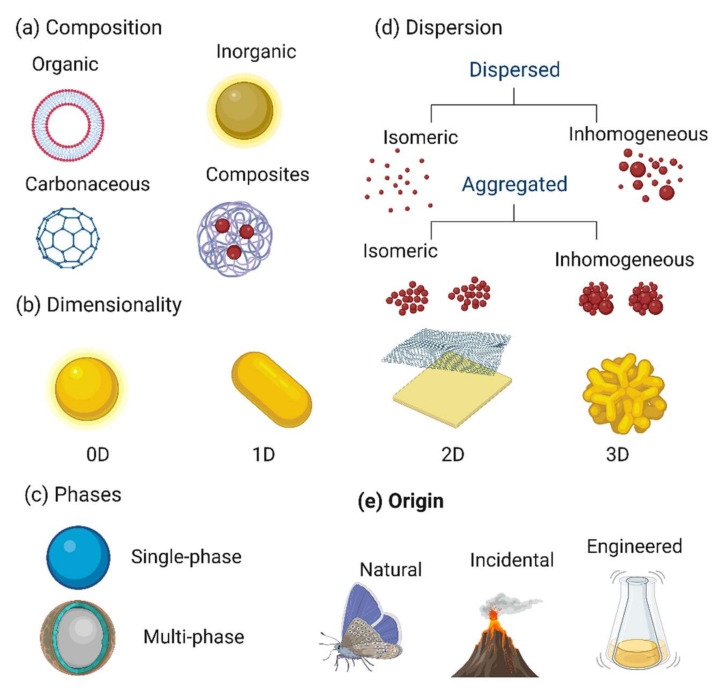
Illustration of how nanomaterials can be classified in function of their composition, dimensionality, phases, dispersion, and origin. Image created with BioRender.

(1) Zero-dimensional (0D) NMs (all three dimensions in the nanoscale; i.e., up to 100 nm) include quantum dots (carbon, graphene, inorganic) and other spherical NMs (noble metals, fullerenes, polymers, metal organic framework, Up- and down-conversion nanoparticles) [22,23]. Due to their chemical inertness, biocompatibility, optical stability, cell permeability, and wavelength-dependent photoluminescence, they are interesting for biomedical and optoelectronic applications [24].
(2) One-dimensional (1D) NMs (one dimension > 100 nm). In this class, nanotubes, nanorods, nanowires, and nanofibers [25] are made of polymer, carbon, metals, and metal oxides and are good electron emitters in a weak electric field. Other 1D NMs, such as veils, mats, and nonwovens, are made of polymer nanofibers [26,27]. Due to their important surface-to-volume ratio and small pores, they are used for filtration, decontamination, and catalysis and as scaffolds and super-absorbents for wound dressing and tissue engineering [28].
(3) Two-dimensional (2D) NMs (two dimensions > 100 nm) include platelet-like forms, graphene (graphene oxide and re-reduced graphene oxide), transition metal dichalcogenides, metal oxides, silicates, graphitic carbon nitride, layered double hydroxides, black phosphorus, tin telluride nanosheets, antimonite, hexagonal boron nitride, boron nanosheets, and other sheet-like NMs [29,30]. Their physicochemical, biological, and optical properties explain their uniform shape, surface charge, and high surface-to-volume ratio [31].
(4) Three-dimensional (3D) NMs (no dimension in the nanoscale range) include nanoporous powders, nanowire bundles, nanotube bundles, nanolayers, and nanostructured electrodes. Much research has been done on the development, fabrication, and evaluation of 3D NMs for storage devices (supercapacitors and batteries) for wastewater treatment and electrochemical conversion [32,33,34]. These complex NMs are important components of biomedical devices, solar cells, microelectromechanical systems, and robotic technology [35]. The use of 3D printing of NMs will allow the development of architectures with improved functional integration [21]. Figure 1 shows different NM classifications in the function of their properties.

### 2.2. Classification Based on Porosity

**Figure 2:**
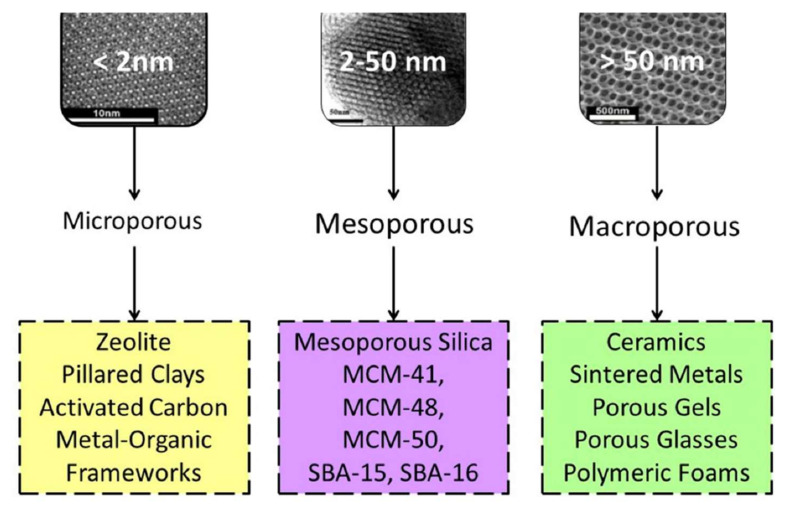
IUPAC classification of NMs in the three indicated classes in the function of their porosity MCM-41, MCM-48, MCM50, SBA-15, and SBA-16 are special types of organized mesoporous silicate systems [10]. Image created with BioRender.

According to the International Union of Pure and Applied Chemistry (IUPAC) nomenclature, NMs can be classified into three groups in the function of their pore size: mesoporous, microporous, and microporous NMs (Figure 2) [36].

(1) Mesoporous NMs are highly porous compounds with pores of 2 to 50 nm in size. Moreover, according to IUPAC, highly permeable matter can be disorganized or organized in different units. Highly porous carbon is permeable in the microporous region, which significantly increases the insert surface area. Adsorbents are popular highly porous compounds that generally consist of a carbon structure with a permeable structure and a micropore volume, depending on how they were prepared [37].
(2) Microporous NMs have pores < 2 nm in diameter and are commonly defined as nanopores (e.g., zeolites and metal-organic frameworks). Microporous materials are widely used for air filtration and gas separation to provide a contaminant-free gas exchange [38]. Mold spores, bacteria, and other air contaminants can be removed, while gas molecules can pass through the micropores. This allows obtaining a sterile environment within an enclosed area [39].
(3) Macroporous NMs have pore sizes > 50 nm (e.g., macroporous arrays) and are particularly interesting due to their enhanced transport properties. Organized macroporous arrays should exhibit optimal fluxes, and diffusion should not be a limiting problem. This is a key issue for all processes where accessibility is crucial, for instance, delivery, sensing, catalysis, and sorption [40].

### 2.3. Classification Based on the Nanomaterial Source

NMs are also characterized as biological, incidental, engineered, or bioinspired materials in function of their origin [10,41].

(1) Natural NMs are materials formed through natural (bio)geochemical or mechanical processes (e.g., combustion materials from forest fires, acid mine drainage, volcanic ash, sea spray, and radioactive radon gas waste) without any direct or indirect contribution by anthropogenic activities and processes [42]. Examples of natural NMs include the blue colors of tarantula, some butterfly wing scales, silk spiders and spider mites, foraminifera, viral structures such as capsids and proteins, wax crystal coating, lotus or nasturtium leaves, gecko foot spatula, natural colloids (milk and blood), human bone matrix, coral, nacre, and horn materials such as feathers, hair, skin, and claws [41,43]. Some inorganic NMs are formed naturally by crystal growth. For example, clays exhibit complex nanostructures due to their anisotropic crystal structure. Opals are probably formed by volcanic activity. Moreover, natural photonic crystals are considered NMs because of their nanoscale structure [44].
(2) Incidental NMs are created unintentionally by direct or indirect human actions (e.g., vehicle engine exhaust, welding gasses, solid fuel combustion, and cooking). Incidental NMs unintentionally formed during an intentional process can increase air pollution. Many NMs (e.g., pigments, fumed silica, and cement) are formed during forest fires [41]. It is difficult to determine when incidental NMs started to be produced by humans. Usually, in incidental NMs, size and shape are not regular. They strongly affect the environment and should be compared to engineered NMs [45].
(3) Engineered NMs are manufactured to fulfill specific needs (e.g., nanostructured medical implants) [46]. These nanoparticles have regular shapes and sizes (rings, fullerenes, carbon nanotubes, spheres, and graphene), whereas natural and incidental nanoparticles have irregular shapes and sizes, such as carbon black [41,47]. In the 1940s, the first commercialized NMs were prepared from fumed silica, and in the 1960s, the first silica nanospheres were fabricated from aqueous solutions [45].
(4) Bioinspired NMs are fabricated to obtain specific nanostructures, features, or functions to mimic natural materials or living organisms. In many bioinspired NMs, advanced nanofabrication techniques are used to modulate their structures and obtain specific functions. For example, the rapid color change observed in chameleons when fighting or during courtship is mainly explained by the lattice adjustment of guanine nanocrystals in iridophore cells [48]. The photonic structure of chameleon iridophores can be mimicked by incorporating silica nanocrystals into mechanochromic elastomer sensors as non-dense packed crystals. These sensors change color when stretched (from red to blue) and when compressed (from red to green). This effect is reversible, as observed in chameleons. Such sensors may be used in wallpaper, signs, and optical records [41,49].

### 2.4. Nanomaterial Classification in the Function of Their Chemical Composition

NMs can be made of one or more periodic table elements, particularly natural and engineered NMs. Therefore, NMs can be classified as carbon, inorganic, organic, or hybrid NMs.

(1) Carbon-based NMs can be produced from sp2 carbon (e.g., fullerenes, graphene, carbon nanotubes, nanohorns, nano-onions, nanographite, nanodiamonds, carbon nanofibers) using various techniques, such as laser ablation, arc discharge, and chemical vapor deposition (CVD) [50,51]. Carbon-based nanoparticles are a special NM type due to their wide range of allotropies and can be considered to be organic NMs due to the presence of C-C bonds. Nanodiamonds, carbon black, and activated carbon (made of non-sp^2^ hybridized carbon atoms) also belong to this category. Milling or seeding can be used to reduce the size of most NMs present in the environment (e.g., CVD for nanodiamonds) [52]. Carbon-based NMs have been playing an important part in human activities (e.g., composites, pigments, reinforcing materials, fuels). In the field of renewable energy, graphite blocks are used as reflectors and moderators in nuclear reactors [14]. Moreover, carbon nanostructures serve as electrodes in electrochemical sensors, rechargeable batteries, and supercapacitors, [53,54].
(2) Organic NMs are mainly made of carbon and hydrogen, with which other elements are chemically associated to obtain NMs with specific functionalities (e.g., dendrimers, micelles, liposomes, and ferritin). Organic NMs also include lipid and polymer nanoparticles that usually have a nano-encapsulated form (10–1000 nm in size) [55]. The polar lipid assemblies at the cell membranes of some bacteria and viruses are called lipid bilayers. These bilayers are mimicked by Langmuir–Blodgett films made of amphiphilic organic compounds in which one polar nanoblock interacts with another polar nanoblock. The head is on the polar side while the tail is on the polar side, and both have the same size [10]. In these fabricated films, the hydrophilic “head” and the hydrophobic “tail” allow the formation of micelles, liposomes, and single or bilayer films. Micelles and liposomes have a hollow core [56,57].
(3) Inorganic NMs are composed of or include non-carbon elements (e.g., metals, metal oxides, and metal salts). Such NMs have many shapes (e.g., cylinders, wafers, ellipses, cubes, spheres, stars) in the function of the atom packing while maintaining the crystalline nature of metal-based compounds [58,59]. In addition, there are amorphous inorganic nanoparticles. Due to the pendulous bonds of atoms, the surface of inorganic NMs is very reactive and sensitive. This drawback can be overcome through functionalization. Some inorganic NMs have remarkable features, particularly metal-based quantum dots (1-10 nm) due to the transition stage between mass and few atoms, and magnetic nanoparticles [e.g., iron (Fe), magnetite (Fe_3_O_4_), and γ-Fe_2_O_3_ [60,61] due to their strong coercive forces and paramagnetic properties [62]. Nanoclays (1nm-thick 2D silicates) are biocompatible and have low toxicity [63]. The main applications of nanoclays are membrane coatings, polymer reinforcement, barriers, toxin adsorption, and sterilizing materials. Zeolite is a non-toxic, nanoporous, hydrated crystalline aluminosilicate with ion exchange properties for the removal of hazardous pollutants from wastewater [13].
(4) Hybrid NMs are multiphase solid materials in which one of the phases has dimensions less than 100 nm [64]. In polymeric nanohybrids, polymers serve as a matrix for organic or inorganic nanoparticles in various forms [65,66]. This class also includes porous media, colloids, gels, and copolymers. Inorganic nanocomposites combine two or more metals in metal nanocomposites, such as intermetallic compounds, alloys with nanometals, core-shell nanoparticles, and banded components [67]. One of the most important nanohybrids is the carbon nanotube-metal matrix composite, an emerging new material being developed to take advantage of high tensile strength and electrical conductivity. Nanohybrids occur in nature, for example in the structure of abalone shells and bones [68,69].

### 2.5. Other Classifications

There are also other, less common, NM classifications in the function of their phase structure [single-phase (metal nanoparticles), two-phase (core-shell nanoparticles) [70], and multiphase (alloy nanoparticles) NMs] and their molecular structure (crystalline, semi-crystalline and amorphous) [71].

## 3. Mechanisms of Nanoparticle Formation

The mechanisms underlying nanoparticle nucleation and growth are a hot topic because they allow greater flexibility and control over nanoparticle composition and fabrication, particularly nanoparticle diameter and randomly oriented molecules [72]. Knowing the nucleation mechanisms is needed for a comprehensive understanding of NM morphology and is the key step for creating systems with the required functionalities [73]. Nucleation initiates the formation of nanostructures (crystalline or amorphous) from a reaction phase (gas, plasma, liquid, solid). Nucleation is the initial process in the formation of a crystal from a solution, liquid, vapor, or solid, in which a small number of ions, atoms, or molecules arrange themselves in a pattern characteristic of a crystalline solid and form a site where additional particles are deposited as the crystal grows [74,75]. Several factors can influence crystal nucleation and growth. Increasing the temperature lowers the critical supersaturation and increases the nucleation rate. Changing the reaction volume affects the nucleation rate, hence the solution volume should remain the same for a given reaction system [46]. The ionic strength affects the activity of the reaction species (ions) and affects the ionic double layer at the crystal interface and crystal growth. The ratio of the ionic species to the stoichiometry of the crystal unit affects the adsorption of the ionic species on the nuclei and subsequent crystal growth. The agitation of highly supersaturated solutions leads to nucleation by shear-induced nucleation or to an increase in fluctuation. Foreign particles or bubbles can induce heterogeneous nucleation [76].

### 3.1. Nucleation

**Figure 3:**
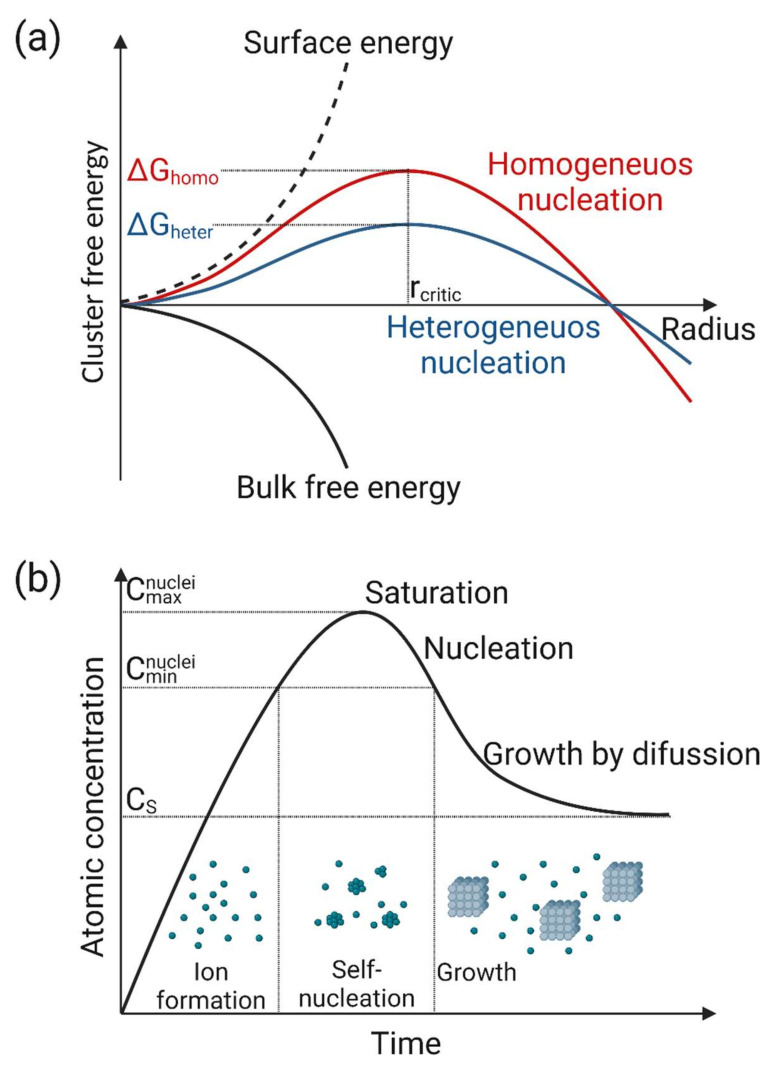
Schematic representation of the nucleation steps (**a**) In the classical nucleation theory, cluster-free energy (ΔG) is influenced by the cluster radius (r). The maximum ΔG is reached at the critical cluster size r_c_ when the first stable particles are formed. (**b**) Nucleation mechanism proposed by La Mer and Dinegar in which nanoparticle formation is shown as a function of time. The image was created with BioRender.

Nucleation requires relatively high supersaturations, and they exhibit a high-order dependence on supersaturation. From a kinetic point of view, the nucleation step is fast and cannot be observed with ordinary electron microscopes. The mechanism of the process includes two steps that are slow, continuous nucleation and fast, autocatalytic surface growth. Primary nucleation mechanisms involve the formation of clusters, i.e., crystalline materials most often existing on the 0–2 nm scale, which is typically a reversible process. When the critical activation energy is reached, an irreversible phase change takes place leading to nucleation [77]. The energy diagram in Figure 3a helps to understand the nucleation process in function of the changes in the surface and bulk-free energies during such process. In general, the nucleus is in a stable state and does not go back to the dissolved gas state [72]. After the critical point is reached, nuclei grow and form a stable nanoparticle. For example, when metallic nanoparticles are fabricated, the metal cation-containing precursor is reduced or decomposed into metal atoms (zero oxidation state) that then combine, leading to nanoparticle formation. To date, different nucleation mechanisms have been observed and reported in the literature. Figure 3b shows the nucleation mechanism proposed by La Mer and Dinegar [78].

**Figure 4:**
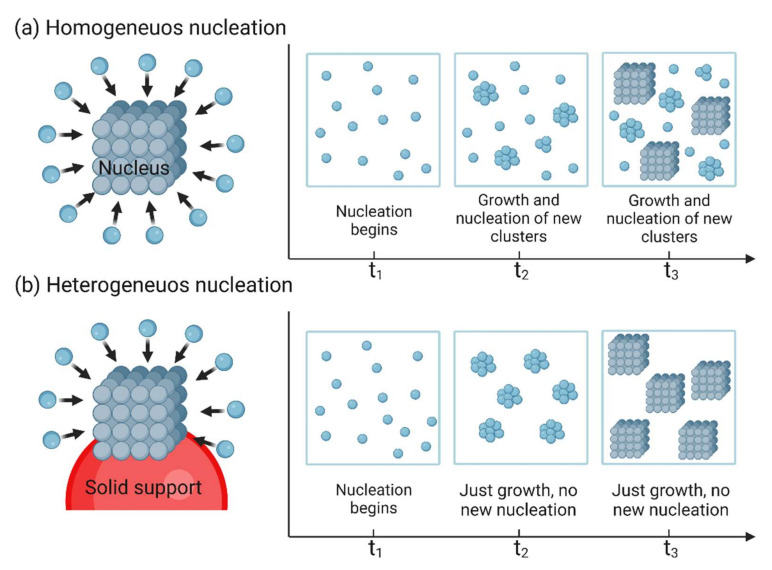
Schematic presentation of (**a**) Homogeneous nucleation and (**b**) Heterogeneous nucleation and reaction coordinates as a function of time. Images were created with BioRender.

Nucleation occurs when a small nucleus begins to form in the liquid, which can occur homogeneously inside the bulk of the liquid or heterogeneously on the surface of solid support [79,80,81]. Heterogeneous nucleation occurs on the surface of impurities or of special additives that can be added. It occurs at active sites, such as phase boundaries, surfaces (e.g., of containers, bottles), and contaminants (e.g., dust). Active nucleation is facilitated by the lower surface energy that reduces the free energy barrier. Therefore, heterogeneous nucleation can occur at driving forces that are much lower than those required for homogeneous nucleation [82,83,84]. In homogeneous nucleation, nuclei develop uniformly from the precursor. The tiny crystalline region in the solidification phase formed in homogeneous nucleation is due to the particle morphological properties. Heterogeneous nucleation is slightly more frequent than homogeneous nucleation. This is in line with the assumption that at favorable sites for heterogeneous nucleation, interfacial vitality may be relatively low. This decreases the initiation force and increases the likelihood of nanoparticle formation at such sites [80]. Figure 4 shows the reaction coordinates of heterogeneous and homogeneous nucleation over time.

### 3.2. Growth

Understanding the growth mechanism of nanoparticles is very important for size and shape control, which is necessary to obtain a reproducible production process. The growth stage then proceeds with the addition of atoms to the growing crystal. For many years, the process of the growth of nanoparticles has been described through the LaMer burst nucleation and following Ostwald ripening to describe the change in the particle size (Figure 3b) [85]. Ostwald ripening occurs because molecules, atoms, or ions on the surface of nanoparticles are more energetically unstable than those in the bulk. Therefore, these unstable species at the nanoparticle surface often go into solution shrinking the larger particle over time and redeposit the dissolved molecules onto the small particles. Several factors such as the synthesis method, reactant concentration, stirring, pH, temperature, time, pressure, and environment greatly influence the growth of nanoparticles [86]. the pH of the solution medium influences the size and texture of the synthesized nanoparticle. The physical synthesis methods require the highest temperature (>350 °C), whereas chemical synthesis methods require a temperature less than 350 °C. The nanoparticle synthesized using plant extract is greatly affected by the length of time for which the reaction medium is incubated. In many environments, a single nanoparticle becomes core-shell nanoparticles quickly by reacting with or absorbing other materials from the medium [87,88]. Surface active molecules (surfactants) possess a remarkable ability to control crystal growth and direct it in a shape- and size-controlled manner [59]. The aggregation can be minimized or avoided with the choice of a suitable surfactant (CTAB) or stabilizer (PVP, citrate, thiols, polymers, etc.) and (pH), temperature, etc. [89].

## 4. Classification of Nanomaterial Synthesis Methods in Function of the Starting Materials

**Figure 5:**
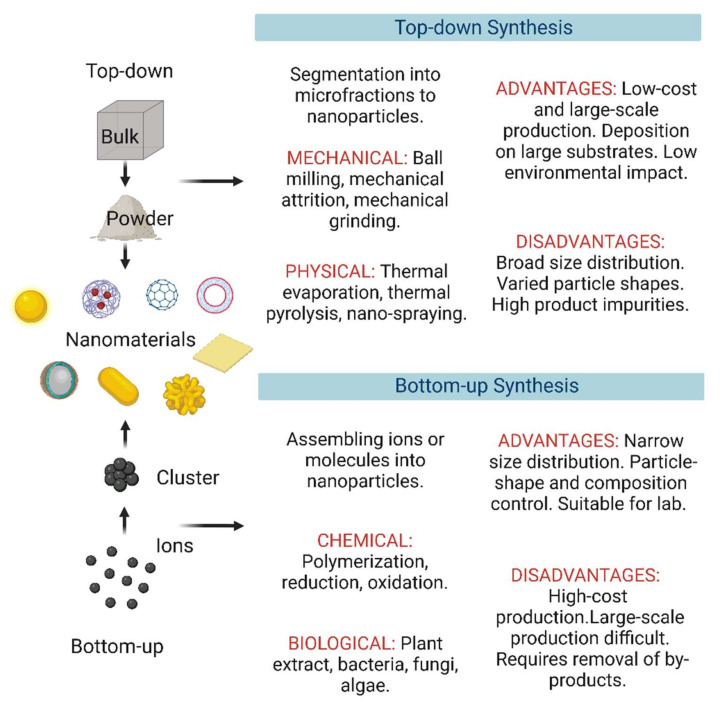
Schematic representation of top-down and bottom-up techniques for nanomaterial fabrication. Figure created with BioRender.

NM synthesis methods can be grouped into three classes in the function of the starting raw materials: top-down, bottom-up, and hybrid approaches (Figure 5).

### 4.1. Top-Down Approaches

Top-down approaches for NM synthesis include mechanical and chemical fabrication techniques. In top-down approaches, NMs are produced by breaking down the bulk material into nanoparticles, typically by attrition, milling, and etching [90]. Top-down approaches are more suitable for fabricating thin films and NMs larger than 100 nm. These techniques are used for the fabrication of electrical circuits with good integration and connectivity [91]. Integrated circuit fabrication is an example of top-down nanotechnology where tiny mechanical components (e.g., levers, springs, and fluid channels) are embedded in a tiny chip. The main drawback of the current top-down methods is their resolution limit [92,93].

### 4.2. Bottom-Up Approaches

Compared with top-down methods, bottom-up methods allow for the creation NMs with fewer faults. In such approaches, atoms, molecules, and nanoparticles are the starting material used to create complex nanostructures [92,94]. Ionic and molecularly self-assembly is a bottom-up approach in which chemical or physical forces are used to gather individual blocks/molecules into larger structures through noncovalent bonds, such as hydrogen and ionic bonds, van der Waals forces, and water-mediated hydrogen bonding [95]. Bottom-up approaches allow spatially controlling the properties and composition of the individual blocks. The building block size depends on the desired properties. Wet chemical techniques (e.g., sol-gel, microemulsion) and co-precipitation are bottom-up approaches [8].

### 4.3. Hybrid Approaches

In hybrid approaches, top-down and bottom-up fabrication techniques are combined to develop nanostructured platforms. These hierarchically organized structures are generally difficult or impossible to fabricate using only top-down or bottom-up techniques [94]. Photolithography is an example of a hybrid approach in which etching is the top-down technique, and layer formation (by growth with ions) is the bottom-up method [96,97].

## 5. Classification of Nanomaterial Synthesis Techniques in Function of the Deriving Forces

Many research organizations proposed to use the originating force nature to characterize NM production processes (Figure 3).

### 5.1. Mechanical Methods

Mechanical processes to separate solid materials into small fractions (e.g., grinding, refining, high-energy ball milling, sequential cutting) are top-down synthesis methods [98]. In mechanical manufacturing (e.g., metallic and ceramic NMs), the microparticle size is reduced by grinding [46]. Specifically, to produce metallic nanoparticles, the starting material (e.g., metal oxide) is ground in high-energy ball mills in which tungsten carbide or steel is the grinding medium. This process requires a lot of energy and occurs in conditions of thermal stress. Moreover, prolonged grinding leads to grinding medium abrasion, and consequently to particle contamination [98]. Chemical or chemical–physical reactions may be associated with mechanical grinding. Compared with the chemical–physical manufacturing processes, comminution by milling results in nanoparticles with a relatively wide particle size range. The particle shape cannot be fully controlled by this method [99]. Mechanical alloying and reactive milling are simple, require few machines, and the material is easy to handle [100]. Their main advantage is that no waste is released into the environment. Conversely, their main disadvantage is that the nanovehicle size cannot be controlled.

The most common mechanical methods are (1) mechanical compaction, followed by metal powder melting and cooling to form nanoparticles from the obtained atomic structures. This allows monitoring and verification [101]; (2) deformation techniques, in which the structure of crystalline materials (e.g., metals, porcelain) is modified to increase the nanoparticle hardness and ductility [102]; (3) milling in which the starting material is pulverized by steel balls to produce powdered nanoparticles of a size range between 3 and 25 nm. This method requires very high energy; (4) scrubbing in which thin silicon strips are rubbed with chemicals (e.g., hydrogen fluoride) to obtain silicone particles on the surface. Then, the strips are immersed in a solution (e.g., isopropanol) and nano-sized droplets are formed using an ultrasonic device [102,103]. Table 1 lists different nanostructure types synthesized using mechanical methods and their diverse applications.

### 5.2. Physical Synthesis Methods

These methods include evaporation, condensation, and laser ablation. Molecule evaporation is followed by rapid programmed condensation to achieve the desired size distribution. Compared with chemical methods, solvent impurities are not detected in thin films synthesized using physical methods, and nanoparticles are uniformly distributed [109]. However, when using a tube furnace at atmospheric pressure for nanoparticle synthesis, a large space is required and also much energy to increase the ambient temperature around the starting material. Moreover, reaching thermal stability is time-consuming [110]. On the other hand, metal (e.g., silver) nanoparticles can be synthesized using a small ceramic heater [111] to vaporize the starting material. Unlike a tube furnace, the temperature gradient at the heating surface is quite steep, and the evaporated vapor can cool down rapidly [112].

Physical vapor deposition (PVD) allows the depositing of inorganic and some organic materials in vacuum conditions. PVD is essentially an evaporative deposition technique in which the material is transferred at the atomic level [113]. This process is similar to CVD, but the starting materials/precursors (i.e., the material to be deposited) are initially in the solid form and not in a gaseous state [114]. Sputtering, electrophoretic deposition, electron beam PVD, pulsed laser deposition, atomic layer deposition, and molecular beam epitaxy are a few of the most studied PVD methods [115]. Thin films produced by PVD are mainly exploited for optical, optoelectronic, microelectronic, and magnetic applications [116]. They are also interesting materials for tribology, thermal insulation, corrosion protection, and decorative coatings [117]. PVD enables the production of a uniform and visible nanoscale coating on the substrate surface. PVD coatings are tough, wear-resistant, and oxidation resistant. However, unpolished are difficult to coat, the capital cost is high, and the deposition rate is quite slow [118].

Spray techniques, such as spray drying, freeze drying, plasma spraying, and hot spraying, are used to synthesize in a single step a wide range of functional, simple, and multicomponent materials [119]. In spray drying, the starting material is atomized into a fine spray mist. During hot air drying, the solvent is vaporized, and the spray mist dries into a solid product in one step. Recently, spray drying technology that focuses on atomization has been developed to fabricate dried nanoparticles to be used as nano-drugs [120]. Spray pyrolysis is a representative thermal “digestion” method for aerosol processing. Spray pyrolysis is often classified as a liquid-phase process because salt solutions, dispersions, emulsions, or brines are used. This method allows producing many types of functional particles and also multi-component materials. Compared with the conversion of gas into particles, the spray method is simple and cheap [121]. In flame spray pyrolysis, a flame is used to generate nanoparticles in the gas phase at high temperatures. Thin oxide films are deposited on a substrate by vaporization and decomposition of the sprayed liquid precursors [122].

Freeze-drying is a physical process in which the solvent is removed from an NM dispersion in three steps: freezing, sublimation drying, and desorption drying. The freezing step is the most crucial phase. The NM dispersion must be frozen rapidly to prevent the formation of large ice crystals that affect the quality of the final product [123]. As various stresses occur during freeze-drying, protective agents may be added to the dispersion to protect the NMs. Similar to freeze-drying, drying with supercritical fluids allows producing nanoparticles with uniform particle size/shape and drug distribution without degradation of the nanoparticles or nanostructures [124,125]. Recently, it has been used to fabricate porous aerogel supports that display good aerodynamic features. Supercritical CO_2_-based technologies allow the fabrication of NMs with different shapes and sizes (e.g., nanoparticles and nanocrystals) by fine-tuning the operating conditions. Compared with conventional techniques, supercritical synthesis (drying) methods require minimal amounts of harmful solvents that are then eliminated from the final product. Therefore, such methods can be used to produce safe NMs as carriers of active ingredients (nano-drugs). Importantly, polymeric carriers can be added as stabilizers to control drug release and improve the active ingredient processability with the chosen technology [126].

Electrospinning is an easy and powerful strategy to fabricate nanofibers and materials with a large surface-to-volume ratio and specific features. Electrospinning is a voltage-controlled process driven by electrohydrodynamic phenomena to generate fibers and particles from a polymer solution [127]. The basic equipment includes a solution in a reservoir (usually a syringe) with a blunt needle (for needle-based electrospinning), a pump, a high-voltage source, and a collector. Key parameters include (1) the polymer molecular weight distribution and architecture; (2) the solution viscosity, conductivity, and surface tension; (3) the electrical potential, flow rate, and concentration; (4) the capillary–collector distance; (5) the chamber temperature, humidity, and air velocity; (6) the collector movement and size; and (7) the needle thickness [128]. This method is used to produce nanofibers starting from polymers (the most used material), metals, and ceramics. Scaffolds can be fabricated by electrospinning nanoparticles mixed with polymers [129]. A wide range of polymers is used for electrospinning, including industrial polymers, biodegradable polymers, specialty polymers, and natural polymers. In general, these polymers should have a high molecular weight and be solvent-soluble [130]. Table 2 lists several nanoparticles, their fabrication method, size, shape, and potential applications.

### 5.3. Chemical and Physicochemical Synthesis Methods

Chemical vapor deposition, sol-gel, solvothermal processes, polymerization, and other chemical precipitation techniques are examples of chemical techniques for the synthesis of NMs [136]. Laser deposition and electrochemical deposition methods are hybrid systems that combine chemical and physical techniques. Electrochemical methods are an example of physicochemical approaches to producing metal nanoparticles in which a metal anode is dissolved in an aprotic solvent [137]. Physicochemical methods include hydrothermal and solvothermal processes, CVD templating, microwave irradiation, combustion, thermal degradation, and pulsed laser deposition [136]. They are usually more complex and hazardous than biological synthesis methods [24]. Indeed, physicochemical methods are expensive and harmful to the environment due to the need for toxic chemicals. These drawbacks limit their application. Wet chemical synthesis can be used to obtain nanoparticles with specific surface morphologies, phases, shapes, and sizes and thus specific properties. Wet chemical synthesis allows fine-tuning the reaction conditions (e.g., temperature, substrate concentration, additives, pH) to obtain the desired nanomaterials. However, the deposition of toxic reaction byproducts on the nanoparticle surface during their synthesis hinders their application in biomedical fields and is an environmental hazard. For this reason, non-toxic biosynthetic methods have been developed [138,139].

Chemical deposition of nanoparticles and thin films is a reaction in which the product self-arranges and coats the substrate. This method can be divided into CVD, chemical bath deposition, and electrochemical deposition. CVD is a deposition process in which chemical reactions occur at the surface of a heated substrate, and reagents are supplied in gaseous form [140]. Then, they chemically react with the other gases to synthesize non-volatile solid thin films on the substrate, which is rarely involved in these reactions. CVD is generally used to fabricate thin nanostructured mixed layers of crystalline inorganic materials, such as ZnS, CuSe, InS, and CdS [141]. In CVD, after monomer deposition, polymerization is initiated in the presence of an initiator (e.g., FeCl_3_) to produce polymers with conductive properties (e.g., polyaniline, polypyrrole, and polythiophene) [142]. CVD allows depositing a vaporized reactant on a surface to form a thin film. Many thin films and NMs can be produced using CVD, particularly graphene [143]. CVD is often used to fabricate solid materials with excellent quality and performance, usually in vacuum conditions. Unlike PVD where the material is deposited by impact in a specific direction, in CVD the material deposition is multidirectional. Other CVD techniques include atmospheric pressure CVD, low-pressure CVD, ultra-high vacuum CVD, plasma enhanced CVD, metal-organic CVD, and photoinitiated CVD [144].

Electrochemical synthesis is a conventional technique for metal nanoparticle synthesis. It takes place at the interface between an electrolyte solution that contains the metal to be deposited and an electrically conducting metal substrate. This method allows the efficient and fast fabrication of highly pure nanostructures (e.g., nanorods, nanowires, nanotubes, nanosheets, dendritic nanostructures) and nanocomposites. In addition, it is cheap and simple, requires low synthesis temperature, is safe for the environment [145], and has high production yields. Electrochemical synthesis techniques also offer the possibility of batch and continuous production. Bimetallic (e.g., Fe–Ni, Fe–Co, and Ni–Pd) nanoparticles can be produced using this method, as well as cobalt nanoparticles with tunable particle size (from 2 to 7 nm) in the presence of tetraalkylammonium salts [146]. Moreover, in electrochemical processes, the final products are not contaminated by byproducts from chemical reducing agents and they can be separated effortlessly from the precipitate. In addition, electrochemical methods permit size-selective particle production. Electrochemical deposition is an attractive and cost-effective approach for the production of thin films that are widely used in the fabrication of printed circuit boards and integrated circuits [147].

Polymerization is used to produce microparticles, nanoparticles, and also thin films of polymers. In this process, smaller molecules called monomers (also known as building blocks) are chemically combined to generate larger molecules or macromolecules [148]. For example, emulsion polymerization uses amphipathic emulsifiers to emulsify hydrophobic monomers in an aqueous phase, and then water- or oil-soluble initiators generate free radicals that initiate polymerization [149]. Emulsion polymerization has been used to prepare inorganic and polymer nanoparticles for various industrial and scientific applications. After emulsification of the hydrophobic polymers in the aqueous phase, water or oil-soluble initiators are used to produce free radicals. Emulsion polymerization allows fabricating polymeric nanoparticles with a high concentration of solid nanoparticles without the need for surfactants [150]. The surfactants used in conventional emulsion polymerization systems must be removed from the final product. However, their removal is often incomplete, is a labor-intensive procedure that increases production costs, requires energy, and has an environmental impact [151,152]. Therefore, emulsion polymerization carried out without emulsifiers (i.e., surfactant-free, emulsifier-free, or soap-free emulsion polymerization) has attracted increasing attention. For example, polymeric nanocolloids with surface functional groups have been prepared to produce carboxylate-polystyrene beads or amidine-polystyrene nanoparticles [153].

Chemical precipitation (or wet precipitation, aqueous precipitation) is particularly interesting for nanoparticle development because it allows the complete precipitation of metal ions. This technique is the most commonly used method to fabricate micro- and nano-particles because it is simple and can produce a wide variety of particle sizes and morphologies. Moreover, it usually results in nanoparticles with a larger surface area [154]. Chemical precipitation and reaction methods are used to produce nano-sized ceramic particles. Briefly, after the addition of a precipitant (e.g., hydroxide, ammonium acid carbonate, or oxalic acid) to the solution containing the cation of the desired oxide, the precipitation products (hydroxides, carbonates, or oxalates) are fired [155]. Chemical precipitation methods include direct precipitation (only one cation in the solution), co-precipitation, and homogeneous precipitation. Well-dispersed yttrium nanopowders have been prepared by precipitating a yttrium nitrate solution using ammonia water and ammonium bicarbonate as precipitants [10,11]. Hydrothermal precipitation is an elementary process and one of the most economical techniques. As the hydrothermal treatment is carried out at high temperatures (80 °C) and for a long time (1.5 h), the deposited nanoparticles are mainly crystalline [156].

Methods based on the use of ionizing radiation to synthesize NMs also have been described. Electron beams, X-rays, gamma rays, and UV light are frequently used for NM synthesis [157]. With this method, metallic nanoparticles can be produced in an aqueous solution in the presence of stabilizers and without chemical reducing agents. Importantly, radiolytic synthesis is a simple production process in aqueous systems that requires minimal amounts of organic solvents and toxic chemicals and in which the final product needs minimal separation and purification [158]. Flores-Rojas et al. (2020) reported that high energy, especially gamma rays (1.17 and 1.33 MeV of ^60^Co), can be used to trigger radical reactions in all three states (solid, liquid, gas). As the absorption of gamma rays is not selective for free radicals and electrons, these radiolytic species in aqueous solutions are often used for the preparation of metallic nanoparticles from their salts. On the other hand, in the production of organic nanoparticles, gamma rays are mainly used to stabilize the synthesized nanoparticles by crosslinking or to initiate the polymerization of monomers that then will form nanoparticles. Therefore, ionizing radiation is particularly interesting for producing a wide range of metallic nanoparticles in which various metal ions, polymers, monomers, and also proteins may be combined [158,159]. Table 3 lists chemical and physiochemical techniques used for the production of nanoparticles and nanostructures and their applications.

### 5.4. Biological or Green Synthesis Methods

Biological synthesis methods include microorganism-assisted, biotemplate-assisted, and plant extract-assisted biogenesis are green synthesis approaches for nanoparticle synthesis [165,166]. Many plant biomolecules, including enzymes, vitamins, polysaccharides, organic acids, amino acids, and proteins, are used for nanoparticle synthesis by plants in a medium enriched with metal ions. The first step involves the preparation of leaf tinctures and the use of biomolecules from plant extracts and various microorganisms, including fungi, bacteria, and lactobacilli. Many active compounds are present in plant extracts, for instance, alkaloids, phenols, terpenoids, quinines, amides, flavonoids, proteins, and alcohols [167,168]. Some of them contribute to the green synthesis of metallic nanoparticles by reducing metal cations to nanoparticles (i.e., reducing agents, such as flavonoids and phenols) and by concomitantly performing stabilization functions to avoid nanoparticle aggregation.

Extraction is a solid–liquid separation process for isolating specific plant components in which the plant components (i.e., the solid object) are dissolved and entrapped in the solvent (i.e., the liquid). The plant extract concentration, pH, temperature, and contact time are known to affect the size, shape, and yield of the nanoparticles. Compared with the other previously described methods, the use of plant extracts for nanoparticle synthesis is particularly interesting because it is less expensive, more environmentally friendly, can be scaled up for industrial applications, and can be performed without high pressure, energy, temperature, or toxic chemicals [169]. Indeed, for nanoparticle synthesis, plant extracts are simply mixed with a metal salt solution at ambient temperature for a few minutes. Any plant part can be used to synthesize nanoparticles, such as leaves, stems, stalks, and flowers. Some of the examples of synthesis of Ag NPs using leaf extract include the use of Cinnamomum camphora, Alfalfa, Persimmon, Magnolia, Cycas, Geranium leaves, Aloe vera, Holarrhena antidysenterica, etc. [170]. Leaf extracts are environmentally friendly, non-hazardous, and non-toxic reducing agents. Research should now focus on whether plant biomass or extracts can be used to fabricate noble metal (silver, gold, platinum, and palladium) nanoparticles with a specific shape and size. Indeed, due to the great plant diversity, it is not known whether they could be useful for the production of such nanoparticles [171].

Microbes also can be used as nanofactories for the green synthesis of nanoparticles with multiple potential applications in medicine, from antimicrobial, antiproliferative, antioxidant, and anticancer agents to drug delivery systems [172]. Microorganisms (e.g., bacteria, yeasts, viruses) can produce many metal and metal oxide nanoparticle types through different steps: metal ion trapping outside or within the microbial cells [173], enzymatic reduction, and capping. As different microorganisms can be used for the synthesis of nanoparticles, this represents a real alternative to more conventional synthesis methods. For instance, the capacity of fungi to produce and release the specific enzymes needed for nanoparticle synthesis can be stimulated with a growth medium that contains the specific substrate for the fungal enzyme that will degrade silver and produce nanoparticles [169]. Yeast cells bearing metal nanoparticles are “green” hierarchical particle systems that do not require chemical stabilizers or dispersants [174,175]. Virus-based nanoparticle synthesis is a simple, non-toxic, and environmentally friendly method. Plant Viruses and Bacteriophages are interesting for nanoparticle production due to their characteristic geometries and uniform size distribution. The capsid proteins of plant viruses can be used to synthesize new NMs and can self-assemble into viruses with modified coat protein subunits and internal and external size. Particularly, the nanomaterial is enveloped by the virus and thus is protected [176]. Table 4 lists some examples of the biological synthesis of nanoparticles and their uses.

## 6. Nanosynthesis Method Classification in the Function of the Reaction Phase

NM synthesis methods can be classified also in the function of the reaction phase (plasma, gas, solid, or liquid).

### 6.1. Gas Phase Synthesis

Gas phase processes include gas evaporation, inert gas condensation, flame-assisted synthesis, explosion wire, sputtering, and laser ablation. Over the years, the advantages of gas phase synthesis methods, compared with other synthesis processes, have become evident. Indeed, gas phase synthesis allows precisely tuning the synthesis parameters to modify the NM size, shape, and chemical composition. Virtually all gas phase synthesis methods follow the same steps: (1) precursor components are suspended in a gas phase, (2) such components are converted into tiny clusters, (3) nanoparticle production from these clusters is facilitated, and finally, (4) the manufactured particles are collected. Most bottom-up gas phase NM synthesis methods are based on a molecular process to generate an inert compound that progresses through homogeneous initialization, condensation, expansion, and agglomeration [182]. Producing NMs in the gas phase has several advantages. Specifically, these methods allow the fabrication of complicated molecular scaffolds. Although liquid and solid phase processes can be mixed, sustainable gas processes are more commonly used for nanoparticle fabrication. Moreover, gas phase synthesis processes are safer than vapor processes because even the finest liquid contains harmful residues of materials or impurities. This is particularly important when producing medical-grade electronics. These impurities can be avoided in vacuum and gas phase systems. In addition, gas phase processes, such as evaporation, high-temperature evaporation, and plasma synthesis, can be used to control permeability, crystallite size, thermodynamic properties, elemental composition, clumping degree, and molecular homogenization.

**Figure 6:**
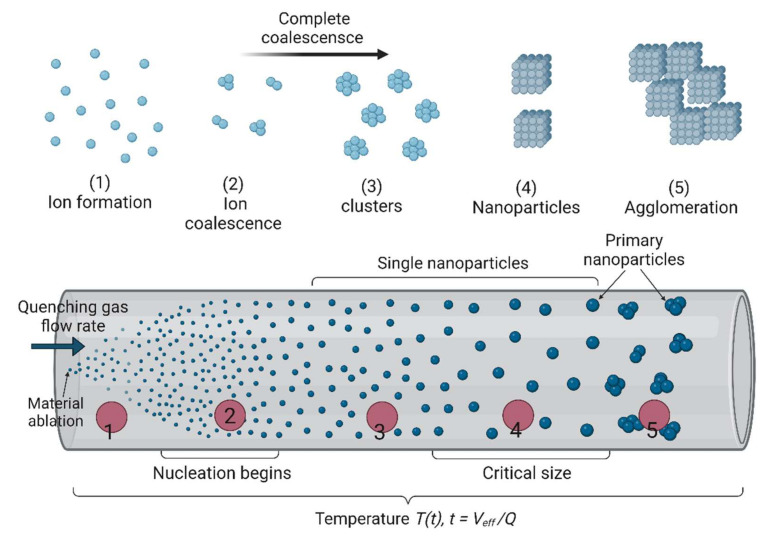
Schematic showing the synthesis of single and agglomerated aerosol nanoparticulates starting from material ablation at atmospheric pressure. Figure created with BioRender.

Gas phase synthesis of nanoparticles has gained interest in recent times, particularly for the large-scale fabrication of nanopowders and different carbon forms (e.g., horns, spheres, wires, sheets, and tubes). When using vapor phase methods for NM synthesis, the vapor phase mixture becomes thermodynamically unstable relative to the solid material to be produced in the nanoparticulate form [183,184]. Figure 6 shows the general approach for the synthesis of high-purity single particles (from single atoms to nanometric particles) by continuous vapor phase synthesis. Vapors are generated by the ablation of localized material with electrical discharges/lasers. Then, such vapors are quenched by an inert gas flow at different temperatures, which leads to particle condensation. The critical core size is lowered to the atomic scale due to the exceptionally high supersaturation achieved in the rapidly quenched vapor cloud, suggesting that particle-particle collision growth may start at the atomic scale. Therefore, particle–particle collision growth is suitable to describe the size changes over time. It should be noted that this model is valid only for rapidly quenched vapors from localized sources [185]. With low quenching currents (and consequently low cooling rates), which is the case for most gas phase nanoparticle production processes, more complicated models are required to predict particle formation and size changes over time. Even at ambient temperature, the atomic clusters and tiny nanoparticles formed at the process start are liquid-like and therefore, can fully fuse into single particles when colliding with other particles. The start of agglomeration (and thus the formation of non-spherical/agglomerated particles) is signaled by the growth of individual particles above the threshold size at which coalescence does not occur any longer or only partially at that working temperature [186].

### 6.2. Plasma Synthesis

Plasma (i.e., a vapor of ionic species) is one of the four basic forms of matter. These building blocks of matter have an electromagnetic potential and at least one remote valence electron (or one added electron). Impurities are removed by liquid-phase and gas-phase processes (e.g., CO_2_, NO_x_, hydrogen chloride) [187]. Due to the extreme heating and dry environment, plasma synthesis enables the production of nanoparticles with distinct and specific surface textures and shapes that cannot be obtained with conventional methods [188]. The value of these particles for industrial technology is determined by their granular properties. PVD with heated plasma could be a much cheaper process for the mass production of NMs compared with the existing gas phase synthesis methods; however, it presents significant logistical challenges [189], such as limited holding time, restricted large computational region, and non-uniform high environment. These issues must be solved to make atmospheric pressure plasma chemical vapor deposition a viable and relatively low-cost method for nanoparticle fabrication [190,191].

**Figure 7:**
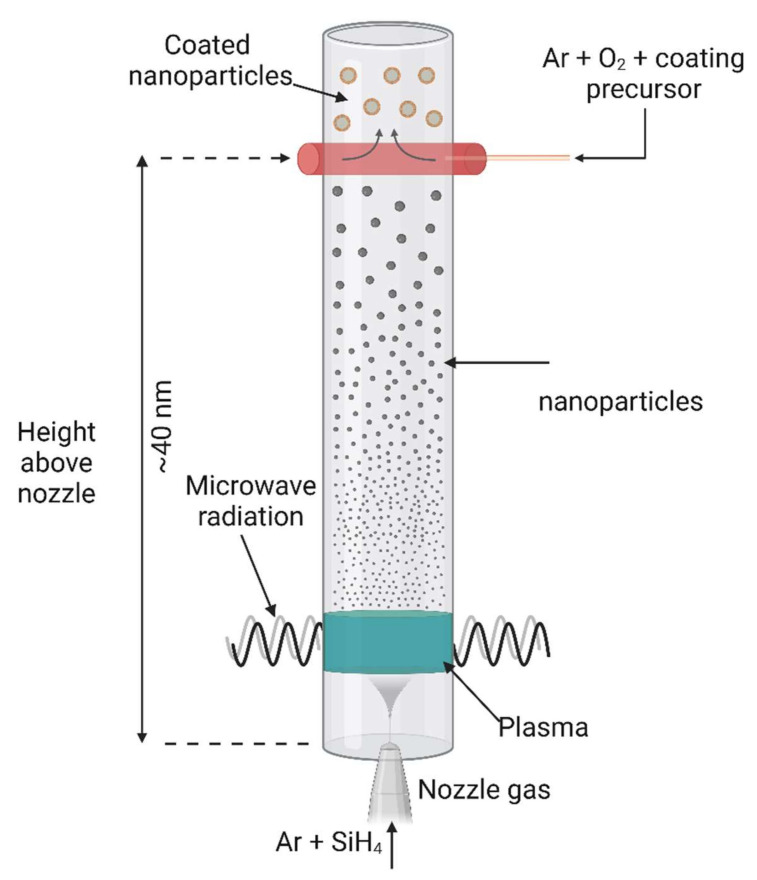
Representation of plasma-assisted gas phase synthesis of silicon-coated nanoparticles. Image created with BioRender.

Figure 7 summarizes the experimental assembly of the reactor for manufacturing silicon nanoparticles by plasma-assisted gas phase synthesis [192]. The reactor includes a quartz tube (70 mm in diameter), a plasma zone, a gas injection system at the bottom, and a coating nozzle (a cylinder with an inner diameter of 70 mm, which corresponds to the quartz tube diameter) below the plasma zone. The plasma in the quartz tube is ignited by a system that combines a cylindrical microwave antenna with annular slots and a 2 kW microwave generator. Plasma gases (Ar and H_2_) and monosilane (SiH4; the silicone precursor), diluted with argon, are coaxially introduced as sheath gas through the central nozzle. SiH_4_ decay in the plasma zone at high temperatures facilitates silicon nanoparticle production. The coating gas mixture, uniformly distributed in the coating nozzle, is injected perpendicularly to the gas flow through 16 holes (0.8 mm in diameter/each) in the nozzle. The coating nozzle shape was designed based on flow simulations to ensure that the exhaust gases (rich in particles) can rapidly mix with the coating gas mixture. Coating sites closer to the plasma zone resulted in precursor pyrolysis and SiC production, while coating sites farther away from the plasma zone resulted in more incomplete precursor degradation [192].

### 6.3. Liquid Phase Synthesis

**Figure 8:**
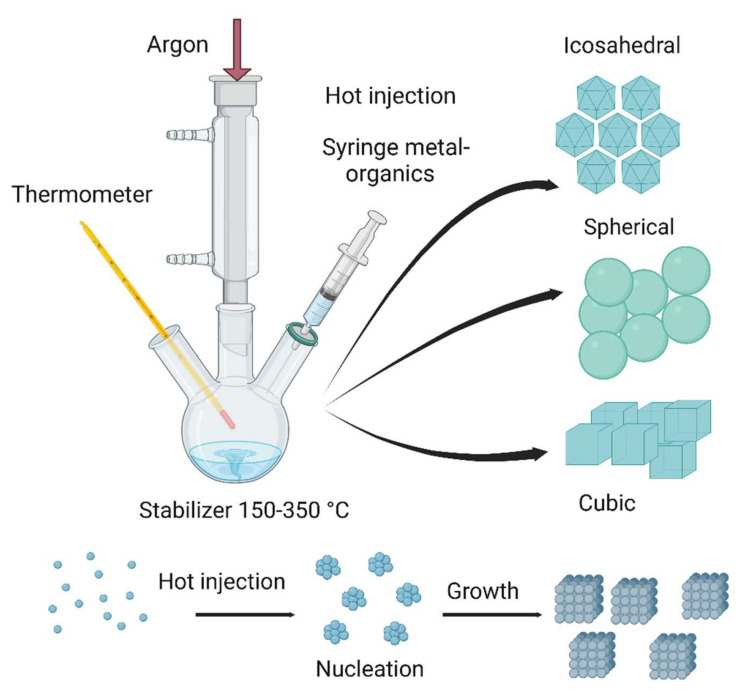
Schematic representation of the hot injection synthesis of nanocrystals. Image created with BioRender.

A series of precisely controlled chemical processes can be used for the bottom-up liquid phase synthesis of nanoparticles with the desired size, morphology, and surface functionality. In addition, self-limiting self-assembly processes have been developed by regulating the growing environment [74]. Indeed, nanostructures can be naturally produced in the liquid phase during the erosion and chemical decay of organic (e.g., plant, microbes) and inorganic (e.g., clays) starting materials [193]. In these dissolution modes, the surface features and chemical modification are crucial for the formation of individual nanoparticles in the given medium. Liquid phase synthesis relies on six commonly used methods (i.e., sol-gel processes, microemulsions, hydrothermal, sonochemical, chemical coprecipitation, and electrochemical techniques) [194]. Importantly, a stabilizing agent must be included to avoid aggregation of the produced nanoparticles. Currently, researchers are interested in unraveling the nanoparticle formation mechanisms and the possibilities offered by mechanochemical engineering to overcome the challenges of solution phase synthesis methods. Figure 8 [195] describes a simple hot injection method to synthesize gallium-palladium (GaPd2) nanoparticles. Briefly, palladium iodide (II) is first dissolved in a mixture of different surfactants and preheated to 120 °C under an argon atmosphere. After 30 min, gallium (III) acetylacetonate is added followed by sonication to fully dissolve the mixture. The mixture and hexamethyldisilazane are then injected into a three-neck flask and heated again to 120 °C. Then, the temperature is increased to 320 °C (2 °C/min), followed by cooling in a water bath. The obtained nanocrystals are rinsed with toluene and ethanol followed by centrifugation at 8000 rpm for 5 min [195].

### 6.4. Supercritical Fluid Synthesis

**Figure 9:**
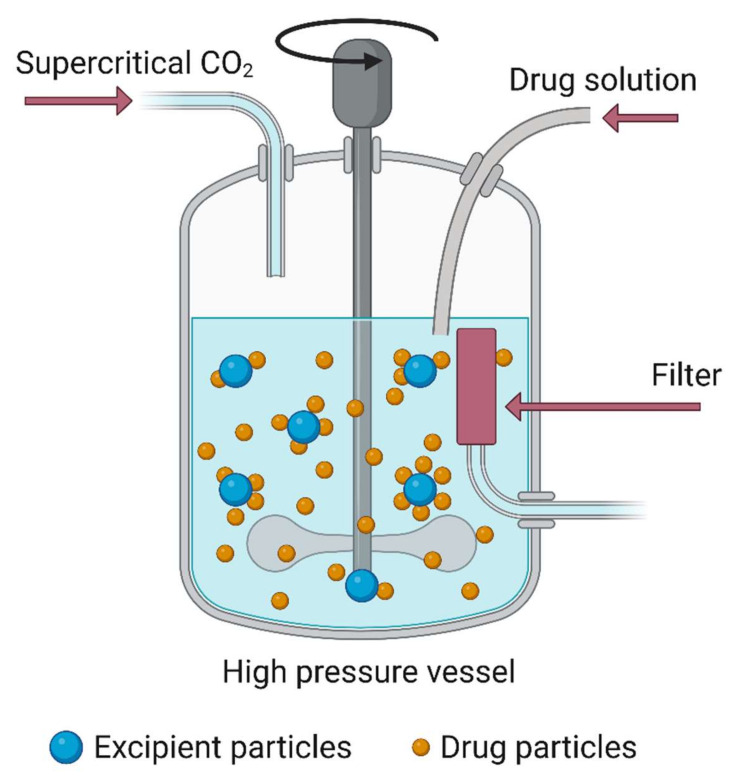
Supercritical fluid technology for producing nanoparticles in which the drug and the polymer (cellulose nanofibers) are processed together to improve the nanoparticle stability and disaggregation. Image created with BioRender.

Supercritical fluids are highly compressed fluids that combine the features of gases and liquids. The supercritical fluid phase is observed when a particular temperature and pressure prevent the gas from condensation into liquid. Supercritical fluid synthesis allows the fast production of high-quality nanocrystals by exploiting the special properties of liquefied gases (solvents) [196]. The main purpose of a chemical transformation in a supercritical medium is to improve the starting material’s physical and chemical processes by exposing compact materials to high temperatures and stress. Chemical processes can be easily controlled with this mechanism. This approach allows the generation of inorganic fission materials, such as iron oxide and nitrates [197,198]. Supercritical fluids are commonly considered “green” solvents because they can be used at moderate temperatures. With supercritical fluid technologies, metal NMs can be continuously prepared, with the possibility of scaling up for mass production. Moreover, micro- or nano-particles with a specific size range can be produced for different applications (e.g., microencapsulation, drug surface coating, co-crystallization with excipients, or host molecules). Supercritical CO_2_ and H_2_O are often employed to produce different NMs (phosphors, magnetic materials, carbon nanomaterials). Figure 9 shows a graphical representation of the supercritical fluid technique used to produce nanoparticles [199] with smooth surfaces, very small size and dispersion, and free flow. However, its disadvantages include the limited dissolving power of CO_2_, high cost, and the need for a large CO_2_ amount. Table 5 summarizes different nanomaterial types synthesized using the supercritical fluid and their applications

### 6.5. Solid Phase Synthesis

**Figure 10:**
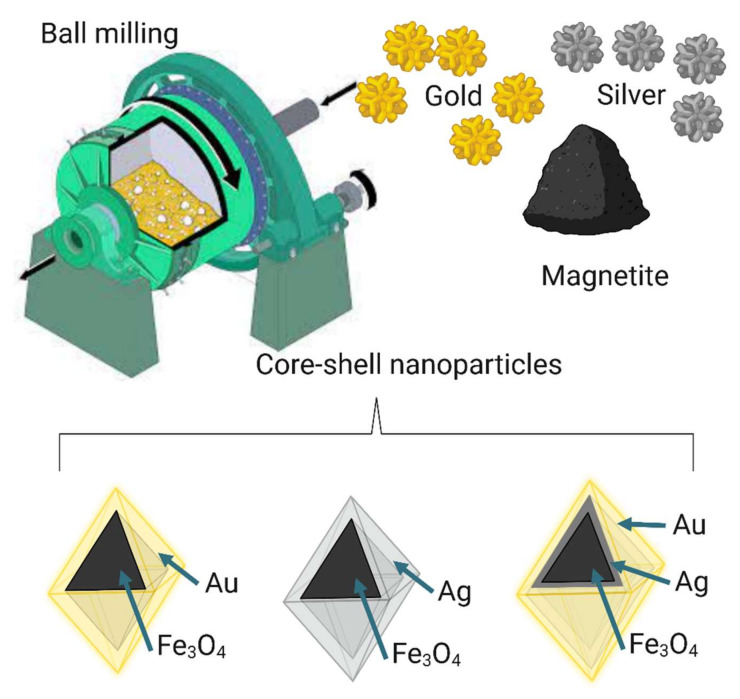
Schematic representation of large-scale solid-state synthesis of Fe_3_O_4_@M nanostructures (M = Au, Ag, or Au-Ag alloy). Image created with BioRender.

Solid-phase nanoparticle fabrication is a top-down technique, unlike liquid- and gas-phase nanoparticle fabrication, which is a bottom-up technique [208,209]. Nalluri et al. (2019) described the fabrication of Fe_3_O_4_@M nanostructures (M = Ag, Au, Au-Ag alloy) using the solid-phase synthesis technique (Figure 10). The metal precursor was vigorously ground over a commercial Fe_3_O_4_ core for 10 min to evenly coat the Fe_3_O_4_ core with the precursor. The resulting mixture was then calcinated at various temperatures for 2 h. An autoclave was used during the metallization process to avoid any surface oxidation. After calcination, the core–precursor mixture was then cooled down naturally in the autoclave furnace [210].

## 7. Concluding Remarks

Engineering NMs with unique properties is of great interest for energetic (generation, conservation, and transformation), environmental (remediation), and health (diagnosis and therapeutics) purposes. This review discussed the different NM classifications (in the function of the origin, composition, size, morphology, porosity, phase, and dispersion state), and synthesis methods (depending on the precursor material, reaction phase, and nature of the derived forces). For nanoparticle synthesis, the possibility of scale-up fabrication is very important. Mechanical methods are already exploited by industries to fabricate nanoparticles for electromagnetic, morphological, and chemical engineering. However, these techniques yield polydisperse, amorphous materials that must be partially recrystallized before they can be combined into nanoscale materials. On the other hand, researchers have not explored yet the potential of liquid phase processes (physical and chemical processes) for the large-scale production of nanoparticles. Other relevant challenges include the cost of intermediates, the recovery of fluids (chemicals and wastewater), and the potentially hazardous effluents. Nevertheless, liquid phase processes may be of interest for commercial high-dimensional research. Gas phase synthesis is a relatively simple method in research laboratories, but more sophisticated processes also are explored. Cost-effective and highly efficient processing and fabrication methods for nanoparticles should be developed to promote their widespread application. Among all gas phase processes, thermal plasma technology is the most promising because it allows the large-scale production of nanoparticles in the gas phase at an affordable cost with minimal waste and hazardous byproducts.

## Figures and Tables

**Table 1:** Mechanical methods used to fabricate different nanoparticle and nanostructure types.

Method	Nanoparticles	Size and Shape	Features/Applications	Refs.
Ball milling	Al nanoparticles	~30 nm, spherical	Quick combustion at a flame temperature of over 1100 °C	[104]
Grinding	NiCoFe–OH Nanoparticles	~15 nm, Nanosheets	Improved electrical conductivity for asymmetric supercapacitor	[105]
High-energy ball milling	P nanoparticles in a carbon matrix	100–300 nm, Carbon fringe	Suitable for large-scale production and better performance in the phosphorus-based anode material	[106]
Mechanical alloying	Al–Y_2_O_3_ Nanocomposites	50–70 nm, spherical Y_2_O_3_	Improved mechanical properties suitable for automotive industries	[107]
Mechanical compaction	TiO_2_ Nanoparticles	~10 nm, Spherical	Improved blending and film forming properties for gas sensing, energy storage, and production	[108]

**Table 2:** Physical methods used to synthesize nanoparticles and nanostructures.

Method	Nanoparticles	Size and Shape	Features/Applications	Refs.
Physical vapor deposition	Magnesium alanate and lithium borohydride	20–40 nm, nanorods 10–40 nm, nanobelts	Improved hydrogen storage	[131]
Pulsed laser ablation	CuO_x_/GrO nanosheets	~60 nm, Spherical (CuO_x_)/nanosheets (GrO)	Enhanced dye removal than graphene oxide	[132]
Spray drying	SiO_2_ nanoparticles modified with alginate	890 nm, smooth doughnut	Drug carrier for cancer treatment	[133]
Solution electrospinning	Chitosan/poly(ethylene oxide)	270 nm, nanofibers	Good mechanical properties with improved properties for drug delivery	[134]
3D printing	Polymethyl methacrylate modified with cellulose nanocrystal-coated Ag nanoparticles	80 nm in width, elongated rods	Antimicrobial biomaterials for functional dental restoration and other biomedical applications	[135]

**Table 3:** Chemical and physiochemical techniques for the production of nanoparticles and nanostructures.

Methods	Nanoparticles	Size and Shape	Applications	Ref.
Chemical vapor deposition	Si nanoparticles	210 nm	Promising for therapeutic and diagnostic applications	[160]
Electrochemical	TiO_2_, nanoparticles	25–30 nm, tetragonal	Provide antibacterial activity against human pathogens	[161]
Chemical precipitation	Pd-loaded on ZnO nanoparticles	40 nm, nanograins	NH_3_ sensing in dry and humid environments	[162]
Hydrothermal	ZnO nanoparticles on porous silicon	20 nm, hexagonal	Improved photo-conversion characteristics	[163]
Radiation	Ni Nanoparticles	~4 nm, Aggregated	Good candidate for energy storage devices as catalysts	[164]

**Table 4:** Biological approaches to synthesize nanoparticles and nanostructures.

Method	Nanoparticles	Size and Shape	Applications/Features	Refs.
Plant extract-based synthesis	ZnO nanoparticles from *Cayratia pedata* leaf extract	52 nm, Spherical	immobilization of the enzyme and bioactive compounds	[177]
Plant extract-based synthesis	Ag nanoparticles from *Plantago lanceolate*	30 nm, Spherical	Antibacterial and antioxidant attributes	[178]
Bacterial synthesis	Au nanoparticles from *Bacillus subtilis*	50 nm, triangular	Drug delivery and biomedical applications	[179]
Fungal synthesis	Pt nanoparticles from *Fusarium oxysporum*	25 nm, Spherical	Potent antimicrobial, antioxidant and photocatalytic activity	[180]
Yeast synthesis	Silver nanoparticles from *Saccharomyces cerevisiae*	16 nm, Oval	Antibacterial applications	[181]

**Table 5:** Different nanomaterial types synthesized using the supercritical fluid and chemical reaction methods for advanced applications.

Nanomaterials	Size	Morphology	Applications	Refs.
Breviscapine-loaded mesoporus silica nanoparticles	177 nm	Spherical shape	Drug delivery for cardiovascular diseases	[197]
Microbeads and nanoliposomes	1 µm (microbeads), 0.2 µm (nanoliposomes)	Spherical shape	Transporters for the delivery of a variety of drugs	[200]
Silicon oxycarbides (SiOC)	2–200 nm	Irregular surface	Improved electrochemical properties for Lithium storage materials	[201]
pH-responsive doxycycline-loaded chitosan nanoparticles	120–250 nm	Spherical shape	Improved adsorption properties for Chemotherapy	[198]
NUFS™-erlotinib nanoparticles	220–250 nm	Round shape	NUFS™-erlotinib more effectively prevents epidermal growth factor receptor (EGFR) signaling and inhibits the proliferation of the non-small cell lung cancer A549 cell line.	[202]
Silk fibroin nanoparticle-decorated poly(l-lactic acid) composite scaffolds	296 nm	Spherical shape	Bone tissue engineering	[203]
Megestrol acetate solid dispersion nanoparticles	500 nm	Spherical shape	Enhance megestrol acetate bioavailability	[204]
Magnetic silk fibroin nanoparticles	75 nm	Spherical shape	Enhance skin permeation (massage-like transdermal drug delivery)	[205]
Salmon calcitonin particles	737 nm	Irregularly shape	Nasal delivery of peptides and proteins	[206]
Valsartan polymer-surfactant composite nanoparticles	400 nm	Spherical	Improve the absorption and bioavailability of weakly water-soluble drugs	[207]
